# Inverse association of serum carotenoid levels with prevalence of hypertension in the general adult population

**DOI:** 10.3389/fnut.2022.971879

**Published:** 2022-09-29

**Authors:** Xu Zhu, Mengshaw Shi, Hui Pang, Iokfai Cheang, Qingqing Zhu, Qixin Guo, Rongrong Gao, Shengen Liao, Yanli Zhou, Haifeng Zhang, Xinli Li, Wenming Yao

**Affiliations:** ^1^Department of Cardiology, The First Affiliated Hospital of Nanjing Medical University, Jiangsu Province Hospital, Nanjing, China; ^2^Department of Cardiology, The Affiliated Suzhou Hospital of Nanjing Medical University, Suzhou Municipal Hospital, Suzhou, China

**Keywords:** serum carotenoids, blood pressure, hypertension, NHANES, WQS

## Abstract

Carotenoid levels are inversely associated with blood pressure (BP). This study focused on the effects of individual and combined serum carotenoids on BP and hypertension, which have not been established to date. Data from National Health and Nutrition Examination Survey (NHANES) 2001–2006 were analyzed in this cross-sectional study. Multivariate logistic, linear, and weighted quantile sum (WQS) regression analyses were applied to explore the associations of six serum carotenoids (α-carotene, β-cryptoxanthin, lutein/zeaxanthin, trans-lycopene, trans-β-carotene, and cis-β-carotene), individually and in combination, with BP/hypertension. The linearity of correlations was further assessed using restricted cubic spline (RCS) regression. A total of 11,336 adults were included for analysis. Data from multivariate models showed that all six carotenoids were independently and negatively associated with both systolic blood pressure (SBP) and diastolic blood pressure (DBP; all *p* < 0.05). Compared to the first quartile, the fourth quartile of α-carotene (odds ratio [OR] = 0.64 [0.52–0.77]), β-cryptoxanthin (OR = 0.74 [0.60–0.90]), trans-β-carotene (OR = 0.50 [0.40–0.61]), and cis-β-carotene (OR = 0.47 [0.35–0.64]) were significantly and inversely related to hypertension (all *p* < 0.05). Moreover, WQS analysis revealed that the combination of all six serum carotenoids was negatively associated with BP and hypertension (all P<0.001), among which trans-β-carotene was the most significant contributor to the protective effect against hypertension (weight, 59.50%). Dose-response analyses demonstrated a linear inverse association of all carotenoids with hypertension (*p* for non-linearity > 0.05). Our collective findings indicate that higher levels of all six mixed serum carotenoids are correlated with decreased prevalence of hypertension, among which β-carotene exerts the most significant effect, which may provide a basis and direction for further studies.

## Introduction

Hypertension, medically defined as systolic blood pressure (SBP) ≥140 mmHg and/or diastolic blood pressure (DBP) ≥90 mmHg, is a leading global public health challenge (2). The prevalence of hypertension has continued to increase over the years. The estimated number of adults with hypertension was recorded as 1.39 billion worldwide in 2010, which is predicted to increase to a total of 1.56 billion by 2025 (3). As a preventable risk factor for various life-threatening conditions, including cardiovascular diseases (CVD), cerebrovascular disorders, and renal failure, inadequate control of hypertension in a timely manner has significant economic and social impacts. The global financial burden of high blood pressure (BP) in 2001 represents about 10% of the world’s overall healthcare expenditure (4). Achievement of effective BP reduction and control is therefore a research direction that warrants substantial attention.

The pathophysiology of hypertension has not been fully elucidated owing to its complexity. However, with progressive understanding of the underlying mechanisms, oxidative stress has been identified as a unifying factor linking complex regulatory systems that sustain the pathophysiology of hypertension (5). A positive relationship between endothelial nitric oxide synthase (eNOS) and a healthy cardiovascular system has been firmly established (6, 7). Previous studies suggest that oxidative stress promotes the uncoupling of NOS, which generates vasoinjurious O_2_^–^ instead of vasoprotective NO. eNOS-derived endothelial NO is an effective regulator of BP oscillations and NO deficiency is reported to cause endothelial dysfunction and hypertension (6). In view of this finding, the relationship between hypertension and antioxidants has attracted considerable research interest.

Carotenoids are a group of antioxidants naturally present in plants, fungi, bacteria, and algae (8, 9). Since the human body cannot synthesize carotenoids, dietary intake is the exclusive source (10). The main carotenoids identified in the plasma of the general population consuming carotenoid-rich foods (fruits and vegetables) include lycopene, α- and β-carotene, lutein, zeaxanthin, and β-cryptoxanthin (11, 12). Ingestion of carotenoids through the diet as well as higher plasma/serum concentrations are reported to reduce the incidence of several chronic diseases (13–15). Moreover, total plasma and serum carotenoid levels <1,000 nmol/L are associated with higher risk of chronic diseases (16). Accumulating evidence supports the protective effects of carotenoids against cardiovascular diseases, including hypertension, a crucial risk factor of CVD (17–19). Recently, the total carotenoid level was shown to be significantly associated with CVD markers, including SBP, pulse wave velocity, Homeostatic Model Assessment of Insulin Resistance (HOMA-IR), blood insulin, triglycerides (TG), and high-density lipoprotein cholesterol (HDL-C) (10). Studies have confirmed a favorable association between carotenoid intake and decreased BP, especially in hypertensive populations (20, 21), which may additionally be beneficial from other perspectives. For instance, the BP-lowering effect of carotenoids is reported to contribute to improved intima-media thickness in adults (22).

Non-pharmacological therapies, including lifestyle improvement and dietary modification, and pharmacological treatments, including antihypertensive, lipid-lowering and antidiabetic medications are the cornerstone of cardiovascular disease prevention. While antioxidants such as carotenoids have not been directly used in the treatment of hypertension, dietary modifications (including the Approaches to Stop Hypertension (DASH) and Mediterranean diets) that recommend regular consumption of carotenoid-rich fruits and vegetables clearly play a role in lowering BP and have been officially endorsed by health organizations (23–25).

Despite considerable evidence in favor of the BP-lowering effect of carotenoids, findings to date are controversial. Thies et al. (26) reported no significant changes in BP after an intervention involving tomato (lycopene) intake in a 16-week single-blinded randomized controlled trial in 2012. Moreover, results on whether carotenoids can lower DBP are inconsistent (21, 27), highlighting the necessity for further evidence supporting the relationship between carotenoids and BP. In addition, the contributions of individual carotenoids in relation to the prevalence of hypertension have not been established.

Herein, we hypothesized that there is a relationship between serum antioxidant micronutrients, combined or individual carotenoids, and the prevalence of hypertension. The National Health and Nutrition Examination Survey (NHANES) is an epidemiological survey with a representative sampling design conducted in the United States. Due to the excellent quality of the survey, this study aimed to determine the associations of serum carotenoid (α-carotene, β-cryptoxanthin, lutein/zeaxanthin, trans-lycopene, trans-β-carotene, and cis-β-carotene) concentrations with the prevalence of hypertension in the general adult population using data from NHANES 2001–2006.

## Materials and methods

### National Health and Nutrition Examination Survey database

The National Health and Nutrition Examination Survey (NHANES),^1^ a major program of the National Center for Health Statistics (NCHS), is designed to assess the health and nutritional status of adults and children in the United States. The survey examines a nationally representative sample of about 5,000 individuals each year and collects detailed medical, dental, and physiological measurements as well as data from laboratory tests administered by highly trained medical personnel for the purpose of health promotion and disease prevention.

### Study population

Data were obtained from NHANES 2001–2006. We enrolled eligible participants with complete data on total and six individual serum carotenoids (α-carotene, β-cryptoxanthin, lutein/zeaxanthin, trans-lycopene, trans-β-carotene, and cis-β-carotene). Exclusion criteria were as follows: (1) lack of three consecutive BP readings, (2) participants aged <18 years, and (3) individuals who were pregnant. All participants provided written informed consent and study procedures were approved by the NCHS Research Ethics Review Board (Protocol Number: Protocol #98-12 and Protocol #2005-06).

### Definition of hypertension

In accordance with most major guidelines and common consensus, this study adopted diagnostic criteria defining hypertension as SPB ≥140 mmHg and/or DBP ≥90 mmHg (2) for analysis as a dependent variable. However, American Heart Association (AHA) and American College of Cardiology (ACC) guidelines for the Prevention, Detection, Evaluation, and Management of Hypertension have recently changed this definition to a lower threshold of 130/80 mmHg, considering the doubled risk of development of coronary heart and stroke in individuals with SBP/DBP of 130–139/85–89 mmHg (23, 28). Thus, we further conducted sensitivity analyses based on the most recent criteria.

### The blood collection procedure

In NHANES mobile examination center laboratories, health evaluations and the collection of fasting blood samples were conducted. Participants in this study who were scheduled for a morning session were required to fast for 9 h. The blood sample collection was dependent on the participant’s age and a minimum of 0.5 ml of serum was preferably taken. On-site centrifugation, aliquoting, and freezing to –70°C were performed on blood samples. The serum samples were transported to central laboratories on dry ice, where they were maintained at –70°C until analysis. The controlled environment of the MEC allowed laboratory measurements to be carried out under comparable settings at each survey site. The procedure on the serum collection and quality control were described elsewhere (29).

### Measurement of serum carotenoid levels

In NHANES 2001–2002 and 2005–2006, serum concentrations of α-carotene, β-cryptoxanthin, lutein/zeaxanthin, trans-lycopene, trans-β-carotene, and cis-β-carotene were measured *via* high-performance liquid chromatography (HPLC). A small volume (100 μl) of serum is combined with an ethanol solution containing retinyl butyrate and non-apreno-β-carotene (C45). The micronutrients are extracted into hexane and vacuum-dried. Insoluble material is filtered from ethanol and acetonitrile-redissolved extract. The filtrate is injected onto a C18 reversed phase column and eluted isocratically with ethanol and acetonitrile. These substances absorb linearly in solution, hence spectrophotometric methods are used for quantitative examination. However, in NHANES 2002–2003, the six serum carotenoids were measured using a comparable HPLC method with multiwavelength photodiode-array absorbance detection at an absorbance of 450 nm. Data from NHANES 2003–2004 were then converted by a regression method to equivalent carotenoids measurements from the HPLC method. Total serum carotenoid concentrations were obtained by summing the concentrations of the six serum carotenoids. Methods for measuring serum carotenoids and quality control were described elsewhere (30, 31).

### Covariate analysis

In the NHANES study, data collection was carried out using a participant questionnaire and medical evaluation. We obtained potential confounding covariates linked to BP or hypertension according to previous findings, including demographic data on age, sex, race, education level, poverty, smoking and drinking status, physical activity, energy intake level, and history of diagnosis of diabetes mellitus. All data were acquired during family interviews with the aid of a standardized questionnaire.

Race and ethnicity were categorized into “Mexican American,” “Other Hispanic,” “Non-Hispanic White,” “Non-Hispanic Black,” and “Other” racial groups. Education levels were classified as “below high school,” “high school,” and “above high school.” Poverty was assessed by poverty-income ratio (PIR) and defined as a PIR < 1 for a family. Participants with serum cotinine values >14 ng/ml were defined as smokers (32). Individuals who consumed at least 12 alcoholic drinks in a single calendar year were considered alcohol users. Physical activity status was classified as vigorous, moderate and inactive. Dietary intake data were collected from two 24 h dietary recall interviews. Intake of energy, nutrients, and other food components was calculated by averaging the 2-day dietary intakes. Measurements of hemoglobin, total cholesterol (TC), and HDL-C in blood samples were obtained from laboratory tests. The estimated glomerular filtration rate (eGFR) was computed using the Chronic Kidney Disease-Epidemiology Collaboration (CKD-EPI) equation (33). Descriptions of each variable are presented in https://wwwn.cdc.gov/Nchs/Nhanes/continuousnhanes/.

### Statistical analysis

Weighted analyses were used to generate accurate national estimates, with adjustment for oversampling of minority subgroups, according to the guidelines of Centers for Disease Control and Prevention (CDC).^2^ Continuous variables were calculated as median (interquartile range) and categorical variables as absolute (percentage) values. The Pearson correlation method was adopted to calculate correlation coefficients for the six serum carotenoids. The concentrations of each of the six carotenoids were log-transformed and divided into quartiles, the lowest quartile being the reference category.

Three sequential multiple linear regression and three multivariate logistic regression models with increasing levels of adjustment for confounding variables were employed to explore the associations of each of the six carotenoids with BP and hypertension. Model 1 was adjusted for age and sex. Model 2 was adjusted as for Model 1 plus race, education level, poverty, smoking, and alcohol usage. Model 3 was based on Model 2 with additional adjustment for physical activity, energy intake level, body mass index (BMI), hemoglobin, TC, HDL-C, eGFR, and diabetes. Further sensitivity analyses using the revised definition of hypertension (SBP ≥ 130 mmHg and/or DBP ≥ 80 mmHg) were performed. A further sensitivity analysis was performed by additionally adjusting our model for dietary intake of individual carotenoids (α-carotene, β-carotene, β-cryptoxanthin, lycopene, and lutein/zeaxanthin), dietary intakes of antioxidant micronutrients (retinol, zinc, selenium, copper, vitamin A, vitamin E, and vitamin C), and serum nutrient biomarkers (serum iron, folate, vitamin B12, vitamin A, vitamin E, and vitamin C).

Considering the significant correlations between serum carotenoids, weighted quantile sum (WQS) regression was performed using the “gWQS” package to assess the associations between levels of the six individual or the mixture of six serum carotenoids and hypertension-related outcomes (SBP, DBP, and prevalence of hypertension), whereby each carotenoid was assigned a weight within the index indicating its contribution to the overall protective association. A linear link was assumed for SBP/DBP and a logitic link for hypertension. Specifically, we established the WQS index of hypertension-related outcomes according to quartiles of serum carotenoid concentrations and applied 40% data as the test set and the remaining 60% as the validation set with a total of 3,000 bootstrap samplings. The WQS regression model was adjusted for age, sex, education level, race, poverty, smoker, alcohol user, physical activity, energy intake level, BMI, hemoglobin, TC, HDL-C, eGFR, and diabetes. Individual serum carotenoid weights of ≥0.1 were considered a significant contribution rate.

A restricted cubic spline (RCS) regression model with three knots (10th, 50th, and 90th percentile) was employed to explore the dose-response relationship between serum carotenoid and prevalence of hypertension. Non-linearity was tested using analysis of variance (ANOVA). All statistical analyses were conducted using R Statistical Software (version 4.1.0^3^), with *p* values <0.05 (two-sided) considered statistically significant.

## Results

### Characteristics of study participants

A total of 31,509 individuals participated in NHANES from 2001 to 2006, 9,331 of whom had missing data on the six serum carotenoids. We subsequently excluded participants lacking three consecutive BP readings, aged <18 years, and those who were pregnant, ultimately leaving 11,336 eligible participants (Supplementary Figure 1).

The survey-weighted sociodemographic and health status characteristics of enrolled participants are shown in Table 1. The weighted study population with 44.0 years median age, was primarily female (50.5%). Median SBP was 119.3 mmHg and median DBP was 71.3 mmHg. A total of 3,649 (28.2%) participants were diagnosed with hypertension.

**TABLE 1:** Survey-weighted, sociodemographic and health status characteristics of adult NHANES 2003–2018 participants with available data (*n* = 11,336).

Variables	Total (*n* = 11,336)
Age, years	44.0 (32.0, 56.0)
Male, %	5,820 (49.5%)
**Education level**, %	
Below high school	3,149 (17.3%)
High school	2,764 (26.0%)
Above high school	5,423 (56.7%)
**Race/ethnicity**, %	
Mexican American	2,418 (7.4%)
Other Hispanic	384 (4.2%)
Non-Hispanic White	5,770 (73.3%)
Non-Hispanic Black	2,331 (10.0%)
Other race	433 (5.0%)
Poverty, %	2,078 (12.2%)
Smoker, %	2,982 (28.8%)
Alcohol user, %	8,042 (74.1%)
Body mass index, kg/m^2^	27.2 (23.8, 31.2)
**Physical activity**	
Never	4,283 (32.4%)
Moderate	3,216 (30.6%)
Vigorous	3,837 (37.1%)
Energy intake, kcal/day	2,079 (1,525, 2,788)
Total cholesterol, mg/dl	196.0 (171.0, 224.0)
HDL-C, mg/dl	1.32 (1.09, 1.6)
Hemoglobin, g/dl	14.6 (13.6, 15.6)
eGFR, ml/min/1.73 m^2^	94.5 (79.5, 108.5)
Diabetes, %	1,027 (6.8%)
Systolic BP, mmHg	119.3 (110.0, 131.3)
Diastolic BP, mmHg	71.3 (64.7, 78.7)
Hypertension	3,649 (28.2%)

Data are presented as median (IQR) or *n* (%); Sampling weights were applied for calculation of demographic descriptive statistics; N reflect the study sample while percentages reflect the survey-weighted; IQR, interquartile range; HDL-C, high-density lipoprotein cholesterol; eGFR, estimated glomerular filtration rate; BP, blood pressure.

### The detection distributions and correlations between serum carotenoids

The baseline distributions and concentrations of the six serum carotenoids are shown in Supplementary Table 1, with the highest mean concentration determined for trans-lycopene (23.82 μg/dl), followed by trans-β-carotene (18.22 μg/dl), lutein/zeaxanthin (15.63 μg/dl), β-cryptoxanthin (9.29 μg/dl), α-carotene (4.36 μg/dl), and cis-β-carotene (1.07 μg/dl). Detailed distribution data on individual serum carotenoids, dietary carotenoids, dietary antioxidant micronutrients, and serum nutrient biomarkers are presented in Supplementary Table 1. The pairwise Pearson correlation coefficients ranged from 0.13 to 0.89, suggesting overall modest to strong correlations between serum carotenoids. The strongest correlation was observed between trans- and cis-β-carotene (*r* = 0.89), followed by trans-β-carotene and α-carotene (*r* = 0.76; Supplementary Figure 2).

### Associations between six serum carotenoids and blood pressure

The results obtained from three multiple linear regression models aimed at evaluating the relationship between serum carotenoids and BP in adults are presented in Table 2. After adjustment for age and sex, α-carotene, β-cryptoxanthin, lutein/zeaxanthin, trans-β-carotene, and cis-β-carotene had significant negative associations with SBP or DBP (all *p* < 0.05), but lutein/zeaxanthin had no association with DBP in Model 1. Following further adjustment for race, education level, smoking, and alcohol usage in Model 2, the results remained stable and statistically significant. In Model 3, all six serum carotenoids were significantly and inversely related to SBP or DBP (all *p* < 0.05) after additional adjustment for physical activity, energy intake, BMI, hemoglobin, TC, HDL-C, eGFR, and diabetes. The collective results confirm significant negative correlations between the six serum carotenoids and BP.

**TABLE 2:** Multiple linear regression associations of serum carotenoids (log2 transformation) with blood pressure in adults.

Carotenoids	Model	Systolic blood pressure		Diastolic blood pressure	
		β (95% CI)	*P*-value	β (95% CI)	*P*-value
α-carotene	Model 1	−1.35 (−1.63, −1.07)	<0.001	−0.28 (−0.47, −0.08)	0.007
	Model 2	−1.30 (−1.59, −1.00)	<0.001	−0.41 (−0.65, −0.17)	0.001
	Model 3	−1.13 (−1.43, −0.84)	<0.001	−0.37 (−0.62, −0.11)	0.007
β-cryptoxanthin	Model 1	−0.98 (−1.34, −0.63)	<0.001	−0.51 (−0.77, −0.25)	<0.001
	Model 2	−1.20 (−1.59, −0.81)	<0.001	−0.64 (−0.92, −0.36)	<0.001
	Model 3	−1.07 (−1.47, −0.66)	<0.001	−0.81 (−1.09, −0.52)	<0.001
Lutein/zeaxanthin	Model 1	−0.97 (−1.58, −0.37)	0.002	−0.09 (−0.55, 0.36)	0.685
	Model 2	−1.16 (−1.78, −0.54)	<0.001	−0.32 (−0.79, 0.15)	0.171
	Model 3	−1.31 (−1.92, −0.69)	<0.001	−0.76 (−1.20, −0.33)	0.001
Trans-lycopene	Model 1	−0.28 (−0.76, 0.19)	0.235	0.72 (0.41, 1.03)	<0.001
	Model 2	−0.04 (−0.50, 0.42)	0.858	0.61 (0.31, 0.91)	<0.001
	Model 3	−0.78 (−1.26, −0.29)	0.003	−0.37 (−0.67, −0.08)	0.016
Trans-β-carotene	Model 1	−1.92 (−2.25, −1.60)	<0.001	−0.64 (−0.86, −0.41)	<0.001
	Model 2	−1.97 (−2.30, −1.64)	<0.001	−0.82 (−1.06, −0.58)	<0.001
	Model 3	−1.76 (−2.09, −1.44)	<0.001	−0.75 (−1.01, −0.48)	<0.001
Cis-β-carotene	Model 1	−2.10 (−2.51, −1.69)	<0.001	−0.73 (−1.05, −0.42)	<0.001
	Model 2	−2.15 (−2.54, −1.74)	<0.001	−0.95 (−1.28, −0.62)	<0.001
	Model 3	−1.85 (−2.25, −1.46)	<0.001	−0.77 (−1.12, −0.41)	<0.001

Model 1 was adjusted as age, sex.

Model 2 was adjusted as model 1 plus race, education levels, smoker, and alcohol user.

Model 3 was adjusted as model 2 plus physical activity, energy intake levels, body mass index, hemoglobin, total cholesterol, high-density lipoprotein cholesterol, eGFR, and diabetes.

Ref: reference; CI, confidence interval; p-t: p for trend.

### Associations between serum carotenoids and prevalence of hypertension

The six serum carotenoids were divided into quartiles, with the lowest quartile being the reference category, and their relationships with hypertension assessed. As shown in Table 3, after log transformation for continuity, all six carotenoids, except trans-lycopene, were negatively associated with prevalence of hypertension. Compared to the reference quartile, quartiles 3 and 4 in Model 1 revealed a protective association between α-carotene, β-cryptoxanthin, lutein/zeaxanthin, trans-β-carotene, cis-β-carotene and prevalence of hypertension. The association remained significant in Model 2 whereby four other covariates were adjusted in order to reduce false positives. However, in Model 3, the protective effect of lutein/zeaxanthin was less significant while data obtained with the other carotenoids were consistent with previous findings. By adjusting Model 3, we found that compared to the lowest quartile, quartile 4 of α-carotene (OR = 0.64, 95% CI: 0.52–0.77, *p* < 0.001), β-cryptoxanthin (OR = 0.74, 95% CI: 0.60–0.90, *p* < 0.001), trans-β-carotene (OR = 0.50, 95% CI: 0.40–0.61, *p* < 0.001) and cis-β-carotene (OR = 0.47, 95% CI: 0.35–0.64, *p* < 0.001) showed significant negative associations with the prevalence of hypertension.

**TABLE 3:** Multiple logistic regression associations of serum carotenoids with hypertension (SBP ≥ 140mmHg and/or DBP ≥ 90mmHg) in adults.

Carotenoids	Log2-carotenoids	Quartiles of serum carotenoids (μ g/dl)				*P*_*trend*_
		Quartile 1	Quartile 2	Quartile 3	Quartile 4	
**α-carotene**						
Model 1	0.80 (0.76−0.83)	Ref (1.00)	0.78 (0.64−0.96)	0.60 (0.51−0.71)	0.45 (0.37−0.54)	<0.001
Model 2	0.80 (0.76−0.84)	Ref (1.00)	0.80 (0.66−0.98)	0.62 (0.52−0.74)	0.46 (0.38−0.55)	<0.001
Model 3	0.88 (0.84−0.93)	Ref (1.00)	0.84 (0.67−1.04)	0.74 (0.62−0.87)	0.64 (0.52−0.77)	<0.001
**β-cryptoxanthin**						
Model 1	0.83 (0.78−0.88)	Ref (1.00)	0.77 (0.62−0.96)	0.62 (0.52−0.74)	0.56 (0.46−0.68)	<0.001
Model 2	0.81 (0.75−0.87)	Ref (1.00)	0.74 (0.59−0.93)	0.59 (0.49−0.72)	0.52 (0.43−0.64)	<0.001
Model 3	0.91 (0.85−0.97)	Ref (1.00)	0.83 (0.66−1.04)	0.72 (0.60−0.87)	0.74 (0.60−0.90)	<0.001
**Lutein/zeaxanthin**						
Model 1	0.82 (0.75−0.91)	Ref (1.00)	0.83 (0.67−1.02)	0.82 (0.67−1.00)	0.69 (0.56−0.87)	0.002
Model 2	0.80 (0.72−0.89)	Ref (1.00)	0.81 (0.65−1.01)	0.79 (0.63−0.98)	0.66 (0.53−0.83)	0.001
Model 3	0.92 (0.83−1.01)	Ref (1.00)	0.93 (0.75−1.15)	0.94 (0.76−1.16)	0.89 (0.72−1.09)	0.276
**Trans-lycopene**						
Model 1	0.96 (0.89−1.03)	Ref (1.00)	0.94 (0.78−1.13)	0.83 (0.72−0.96)	0.92 (0.75−1.14)	0.271
Model 2	0.98 (0.92−1.05)	Ref (1.00)	0.98 (0.81−1.19)	0.91 (0.76−1.00)	0.94 (0.79−1.19)	0.524
Model 3	0.97 (0.90−1.04)	Ref (1.00)	0.98 (0.80−1.21)	0.88 (0.75−1.04)	0.96 (0.77−1.19)	0.463
**Trans-β-carotene**						
Model 1	0.74 (0.70−0.77)	Ref (1.00)	0.71 (0.62−0.82)	0.55 (0.46−0.66)	0.35 (0.29−0.42)	<0.001
Model 2	0.73 (0.69−0.77)	Ref (1.00)	0.71 (0.61−0.83)	0.54 (0.44−0.66)	0.34 (0.27−0.41)	<0.001
Model 3	0.82 (0.77−0.87)	Ref (1.00)	0.82 (0.70−0.97)	0.68 (0.55−0.83)	0.50 (0.40−0.61)	<0.001
**Cis-β-carotene**						
Model 1	0.70 (0.65−0.75)	Ref (1.00)	0.77 (0.59−1.02)	0.54 (0.42−0.71)	0.35 (0.27−0.46)	<0.001
Model 2	0.69 (0.64−0.74)	Ref (1.00)	0.76 (0.58−0.99)	0.53 (0.40−0.70)	0.33 (0.25−0.44)	<0.001
Model 3	0.80 (0.73−0.86)	Ref (1.00)	0.79 (0.58−1.09)	0.63 (0.46−0.84)	0.47 (0.35−0.64)	<0.001

Model 1 was adjusted as age, sex.

Model 2 was adjusted as model 1 plus race, education levels, smoker, and alcohol user.

Model 3 was adjusted as model 2 plus physical activity, energy intake levels, body mass index, hemoglobin, total cholesterol, high-density lipoprotein cholesterol, eGFR, and diabetes.

Ref: reference; CI, confidence interval.

In terms of sensitivity analysis, consistent results were observed when further adjusting our final model for intake of dietary carotenoids (α-carotene, β-carotene, β-cryptoxanthin, lycopene, and lutein/zeaxanthin), dietary antioxidant micronutrients (retinol, zinc, selenium, copper, vitamin A, vitamin E, and vitamin C) and serum nutrient biomarkers (serum iron, folate, vitamin B12, vitamin A, vitamin E, and vitamin C), validating significant inverse associations of α-carotene, β-cryptoxanthin, trans-β-carotene, and cis-β-carotene with prevalence of hypertension (Supplementary Table 2). Similar results were obtained with multiple logistic regression analyses conducted using the more recent definition of hypertension (SBP ≥ 130 mmHg and/or DBP ≥ 80 mmHg; Supplementary Table 3).

### Weighted quantile sum regression analysis of the negative relationship between serum carotenoids and hypertension prevalence

**FIGURE 1:**
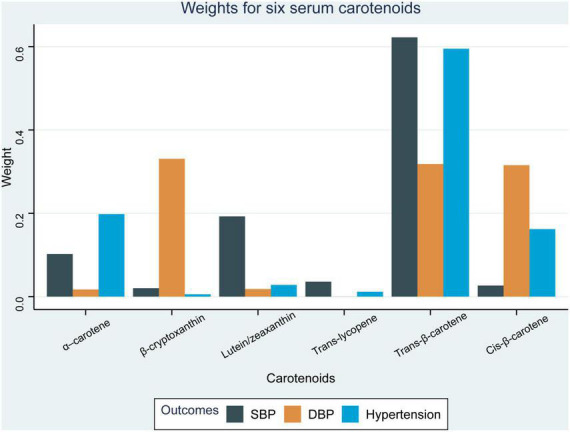
Weights from weighted quantile sum regression (WQS) for the mixture of six serum carotenoids in relation to hypertension-related outcomes. Models are adjusted for age, sex, education level, race, poverty, smoker, alcohol user, physical activity, energy intake level, body mass index, hemoglobin, total cholesterol, high-density lipoprotein cholesterol, eGFR, and diabetes.

Weighted quantile sum regression analysis was applied to assess the inverse association between the six combined serum carotenoids and hypertension-related outcomes (Table 4). The mixture of six serum carotenoids was negatively associated with SBP (β =−2.05, 95% CI: [−2.53, −1.57], *p* < 0.001), DPB (β = −0.73, 95% CI: [−1.04, −0.41], *p* < 0.001), and hypertension (OR = 0.83, 95% CI: 0.77–0.89, *p* < 0.001). The weights of contribution of the individual serum carotenoids to the combined protective effect were further analyzed (Figure 1). Trans-β-carotene had the highest weight in relation to SBP (62.24%) and hypertension (59.50%), respectively, while β-cryptoxanthin exerted the greatest contributory effect on DBP (33.08%). Specifically, α–carotene (10.23%) and lutein/zeaxanthin (19.25%) had weights of >0.1 within the combined effect on SBP while trans-β-carotene (31.81%) and cis-β-carotene (31.55%) had weights of >0.1 in the effect on DBP. α–Carotene (19.80%) and cis-β-carotene (16.20%) had weights of >0.1 in the combined effect on hypertension. Consistent results were obtained upon repeating the analysis using the AHA definition of hypertension. The mixture of all six serum carotenoids exhibited a significant inverse association with hypertension (OR = 0.83, 95% CI: 0.78–0.89, *p* < 0.001), among which the contribution of trans-β-carotene to the protective effect was the most significant (79.10%).

**TABLE 4:** WQS regression model to assess the protective association of the mixture of six serum carotenoids with hypertension-related outcomes.

Outcomes	β /OR	95% CI	*P*-value
Systolic blood pressure	–2.05	(−2.53, −1.57)	<0.001
Diastolic blood pressure	–0.73	(−1.04, −0.41)	<0.001
Hypertension	0.83	(0.77, 0.89)	<0.001

WQS regression model was adjusted as age, sex, education level, race, poverty, smoker, alcohol user, physical activity, energy intake levels, body mass index, hemoglobin, total cholesterol, high-density lipoprotein cholesterol, eGFR, and diabetes. OR, odds ratio; CI, confidence interval; WQS, weighted quantile sum.

### Restricted cubic spline analysis of the association between six serum carotenoids and prevalence of hypertension

**FIGURE 2:**
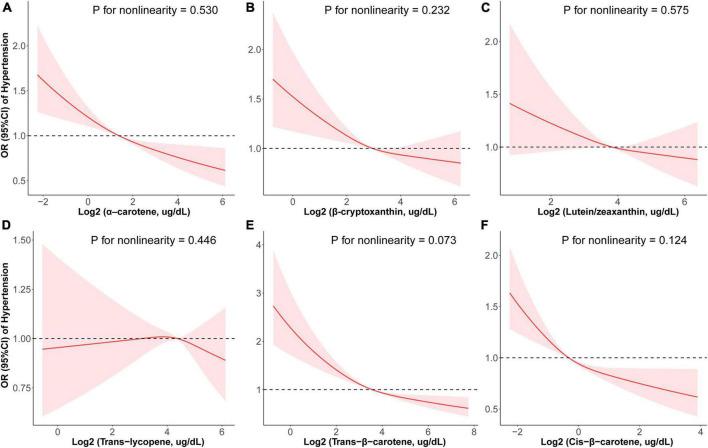
Restricted cubic spline (RCS) analysis with multivariate-adjusted associations between six serum carotenoids and prevalence of hypertension (SBP ≥ 140 mmHg and/or DBP ≥ 90 mmHg) in adults. Models are adjusted for age, sex, education level, race, poverty, smoker, alcohol user, physical activity, energy intake level, body mass index, hemoglobin, total cholesterol, high-density lipoprotein cholesterol, eGFR, and diabetes.

To visualize dose-response relationships between the six serum carotenoids and prevalence of hypertension, RCS regression with multivariate-adjusted associations was adopted (Figure 2). Revise and replace it with [All six carotenoids were negatively associated with prevalence of hypertension in a linear manner (α-carotene: *p* for nonlinearity = 0.530, Figure 2A; β-cryptoxanthin: *p* for nonlinearity = 0.232, Figure 2B; lutein/zeaxanthin: *p* for nonlinearity = 0.575, Figure 2C; trans-lycopene: *p* for nonlinearity = 0.446, Figure 2D; trans-β-carotene: *p* for nonlinearity = 0.073, Figure 2E; cis-β-carotene: *p* for nonlinearity = 0.124, Figure 2F)]. In terms of sensitivity, trans-β-carotene had a non-linear inverse relationship with hypertension (*p* for non-linearity = 0.020) while associations between the five other carotenoids and hypertension were linear (*p* for non-linearity > 0.05; Supplementary Figure 3).

## Discussion

The collective findings of this study clearly demonstrate a significant negative correlation between the six serum carotenoids and BP in the general adult population investigated using the NHANES database (2001–2006). Significant inverse relationships between α-carotene, β-cryptoxanthin, trans-β-carotene, cis-β-carotene and prevalence of hypertension were revealed in our final model. Furthermore, WQS analyses illustrated a negative correlation between the combination of α-carotene, β-cryptoxanthin, lutein/zeaxanthin, trans-lycopene, trans-β-carotene, cis-β-carotene, and hypertension, with trans-β-carotene identified as the most significant contributor to this relationship. In addition, the inverse relationships between serum carotenoids and hypertension were linear.

Our data are consistent with several earlier studies. In a sample of 415 community-based individuals aged between 60 and 64 years, β-carotene was shown to be significantly associated with hypertensive status after taking into account a range of health risk and lifestyle factors that impact BP (20). Another recent study using data from dietary recall interviews suggested that dietary intake of β-cryptoxanthin, lycopene, lutein with zeaxanthin, and total carotenoids were significantly and inversely associated with hypertension risk. Experiments from the current study were different from this earlier report in that we were able to fill in the gaps regarding lack of analyses on circulating carotenoids and avoid recall bias (34). Notably, our results remained largely unchanged after taking dietary carotenoids and antioxidant micronutrients into account. A longitudinal analysis by Hozawa et al. (35) demonstrated that combinations of all serum carotenoids (α-carotene, β-carotene, lutein/zeaxanthin, and cryptoxanthin), except lycopene, were generally inversely related to hypertension, in keeping with our findings. In their study, a −0.09 mmHg decrease in average SBP per 25 μg/L of the baseline sum of four carotenoids (α-carotene, β-carotene, lutein/zeaxanthin, and cryptoxanthin) was predicted (35). Our findings add to the results of Hozawa, providing the specific weights of the contributions from different carotenoids to this overall protective effect. In 2019, Wolak and co-workers (27) conducted an 8-week double-blinded, randomized, placebo-controlled study on 61 untreated hypertensive patients. Data obtained from five groups treated with different doses of lycopene or tomato nutrient complexes indicated effective long-lasting SBP-lowering qualities of lycopene at a dose of 15 mg. However, DBP was not significantly different from baseline with any of the treatments (27). In our analyses, DBP was inversely related to the levels of all carotenoids, excepting lutein/zeaxanthin, with β-cryptoxanthin making the most significant contribution to the combined effect of all six carotenoids on DBP. Our findings are in concordance with a previous report that short-term daily oral supplementation of carotenoid-rich tomato extract induces a significant decrease in SBP as well as DBP (21). Consistently, a study by Chen et al. (36) reported inverse correlations of serum levels of α-carotene and β-carotene with risk of hypertension and SBP.

In the context of hypertension, oxidative stress may trigger reactive oxygen species (ROS) and redox signaling, causing endothelial damage, vascular dysfunction, cardiovascular remodeling, renal dysfunction, sympathetic nervous system excitation, immune cell activation, and systemic inflammation (5). The antioxidant activities of carotenoids have been widely investigated for decades. The biological mechanisms underlying the protective role of carotenoids on hypertension are largely rooted in their ability to quench free radicals, reduce ROS-induced damage, and inhibit peroxidation and scavenge lipid peroxyl radicals (37, 38). Moreover, carotenoids, such as lycopene, are capable of improving the serum lipid level and reducing endothelial dysfunction, which are critical events in the pathophysiology of hypertension (39, 40). Carotenoids also exert positive effects on inflammation by inhibiting lipoxygenase activity, C-reactive protein, and inflammatory cytokines, which could contribute to prevention of insulin resistance (37, 41).

Although extensive research has focused on how carotenoid-abundant food and serum carotenoid levels affect BP and hypertension prevalence, previous studies are associated with a number of limitations. Only certain forms of tomato-related products are proposed to exert a BP reduction effect. Insufficient data are available on the specific components of carotenoids that are most influential in effects on hypertension and potential multicomponent synergistic effects cannot be overlooked (35, 42). To our knowledge, no reports have investigated the specific contributions of individual carotenoids to this protective relationship and the current study is one of the first to fill this gap in the literature. Our data indicate that trans-β-carotene has the greatest weight in the combined protective effect of the six serum carotenoids on hypertension. β-Carotene is an important antioxidant in cardiovascular health. Since trans-β-carotene and cis-β-carotene have different molecular structures, with trans-β-carotene having 100% activity and cis-β-carotene having 53% activity, trans-β-carotene seems to be more bioavailable than cis-β-carotene (43, 44). Dietary intake and/or high blood concentrations of β-carotene are reported to decrease coronary heart disease, stroke, cardiovascular disease, cancer, and/or all-cause mortality (45–47). However, further research is necessary to validate the conclusions drawn from our findings.

Among the carotenoids identified so far, lycopene is the most extensively studied. Several meta-analyses showing that lycopene supplementation has a significant SBP lowering effect (42, 48, 49). Our findings suggested a significant negative association between serum lycopene and BP; however, further analysis of the relationship between serum lycopene and the prevalence of hypertension did not yield consistent results. A similar finding was reported previously (35), which could be partly explained by the epidemiologic discovery that lycopene concentration is positively related to mean intake of meat, alcohol, and serum TG (50).

Our study has several strengths that should be mentioned. First, this is a preliminary evaluation of the contribution of individual carotenoids to the overall protective effect of carotenoids on hypertension. The greatest contributory effect was exerted by trans-β-carotene, which may provide a basis and direction for further studies. Second, our findings are based on a large representative sample of the US general population and therefore have statistical significance. Third, WQS regression was selected in our study since it conserves statistical power and avoids unstable regression coefficients, which may otherwise occur if the highly correlated serum carotenoids are included simultaneously in traditional regression models. Moreover, this approach sheds light on exposure-outcome correlations as well as correlations between exposures.

As with all studies, the limitations of this investigation should be taken into consideration. The causality between serum carotenoid levels and hypertension is not well explored because the temporal relationship between these factors cannot be determined. Furthermore, carotenoids are mostly obtained for humans through fruits and vegetables. Other dietary nutrients, such as dietary fiber, folic acid, vitamins A, C, D, and B12 (51–54), may still have an effect on our findings even after we adjusted our models for confounders associated with hypertension. In addition, the constraints of WQS regression analysis could influence our conclusion. In WQS regression, exposures are constrained to exert the same effect on dependent variable (all positive or all negative).

## Conclusion

After adjustment for age, sex, race, education level, poverty, smoking, alcohol usage, physical activity, energy intake level, BMI, hemoglobin, TC, HDL-C, eGFR, and diabetes, six serum carotenoids (α-carotene, β-cryptoxanthin, lutein/zeaxanthin, trans-lycopene, trans-β-carotene, and cis-β-carotene) concentrations are generally inversely associated with BP, and with the exception of lycopene and lutein/zeaxanthin, similar correlations are evident between serum carotenoid concentrations and the prevalence of hypertension. Our results support a negative relationship between the six combined serum carotenoids and prevalence of hypertension, with β-carotene identified as the most significant contributor to the overall protective effect. Moreover, inverse linear correlations exist between the six serum carotenoids and hypertension. Thus, six serum carotenoids rich in fruits and vegetables may play a role in the beneficial association with elevated BP. Further research is warranted to elucidate the complex interactions among multiple serum carotenoids and their specific effects on hypertension.

## Data availability statement

The datasets presented in this study can be found in online repositories. The names of the repository/repositories and accession number(s) can be found below: https://wwwn.cdc.gov/nchs/nhanes/.

## Ethics statement

The studies involving human participants were reviewed and approved by all participants provided written informed consent and study procedures were approved by the National Center for Health Statistics Research Ethics Review Board. The patients/participants provided their written informed consent to participate in this study. Written informed consent was obtained from the individual(s) for the publication of any potentially identifiable images or data included in this article.

## Author contributions

XZ: conceptualization, methodology, software, formal analysis, writing – original draft, and visualization. MS: conceptualization and methodology. HP and RG: writing – review and editing. IC: formal analysis and data curation. QZ: formal analysis and methodology. QG: data curation and writing – review and editing. SL and YZ: project administration and writing – review and editing. HZ: conceptualization, methodology, writing – review and editing, and supervision. XL: conceptualization, methodology, project administration, writing – review and editing, and supervision. WY: conceptualization, methodology, and data curation. All authors contributed to the article and approved the submitted version.

## Footnotes

1
https://www.cdc.gov/nchs/nhanes/index.htm

2
https://wwwn.cdc.gov/nchs/nhanes/tutorials/default.aspx

3
http://www.r-project.org/
