# Supplementary material for: Inverse association of serum carotenoid levels with prevalence of hypertension in the general adult population
Source: Front Nutr. 2022 Sep 29;9:971879. doi: 10.3389/fnut.2022.971879 (PMC9563225; doi:10.3389/fnut.2022.971879)
Supplement: Supplementary file 1 [file Data_Sheet_1.pdf]

Inverse association of serum carotenoid levels with prevalence of hypertension in the general adult population

Running title: Serum carotenoids and hypertension

Xu Zhu [1†]; Mengsha Shi [1†]; Hui Pang [1]; Iokfai Cheang [1]; Qingqing Zhu [1]; Qixin Guo [1]; Rongrong Gao [1]; Shengen Liao [1]; Yanli Zhou [1]; Haifeng Zhang [1,2,*]; Xinli Li [1,*]; Wenming Yao [1,*]

† These authors contributed equally to this work.

Department and institution of origin:

[1]Department of Cardiology, The First Affiliated Hospital of Nanjing Medical University, Jiangsu Province Hospital, Nanjing, 210029, China.

[2] Department of Cardiology, The Affiliated Suzhou Hospital of Nanjing Medical University, Suzhou Municipal Hospital, Suzhou, 215002, China.

[*]**Corresponding author:**

Wenming Yao, MD, PhD

Department of Cardiology, The First Affiliated Hospital of Nanjing Medical University, Jiangsu Province Hospital, Nanjing, 210029, China.

Email: yaowenming1st@163.com

Xinli Li, MD, PhD

Department of Cardiology, The First Affiliated Hospital of Nanjing Medical University, Jiangsu Province Hospital, Nanjing, 210029, China.

Email: xinli3267@njmu.edu.cn

Haifeng Zhang, MD, PhD

Department of Cardiology, The First Affiliated Hospital of Nanjing Medical University, Jiangsu Province Hospital, Nanjing, 210029, China.

Department of Cardiology, The Affiliated Suzhou Hospital of Nanjing Medical University, Suzhou Municipal Hospital, Suzhou, 215002, China.

Email: haifeng_zhang@163.com

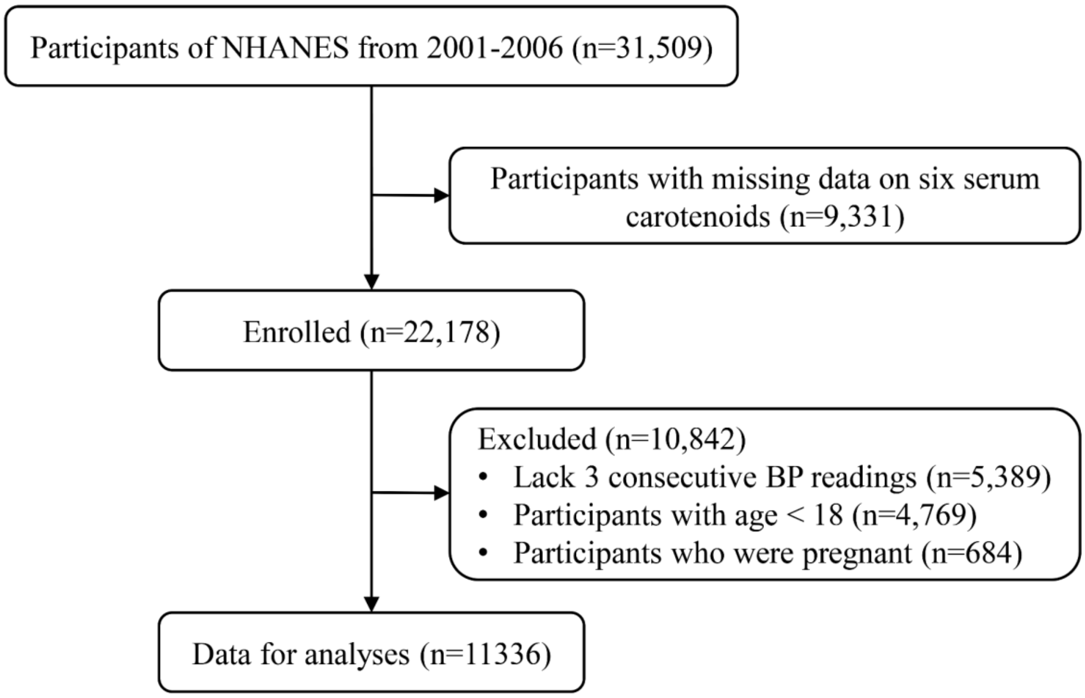

Supplemental Figure 1. Eligible participants in the evaluation of the influence between six serum carotenoids and the prevalence of hypertension in the general adult population.

Supplemental Table 1. Distributions and concentrations of serum carotenoids, dietary carotenoids, dietary antioxidant micronutrients and serum nutrient biomarkers.

Variables	N	Mean	5th	25th	50th	75th	95th
Serum carotenoids							
α–carotene, μg/dL	11336	4.36	0.50	1.42	2.70	5.09	13.20
β-cryptoxanthin, μg/dL	11336	9.29	2.40	4.70	7.21	11.40	23.00
Lutein/zeaxanthin, μg/dL	11336	15.63	6.00	9.80	13.80	19.30	31.40
Trans-lycopene, μg/dL	11336	23.82	7.60	15.70	22.69	30.59	43.70
Trans-β-carotene, μg/dL	11336	18.22	3.30	7.20	12.00	21.70	52.67
Cis-β-carotene, μg/dL	11336	1.07	0.40	0.49	0.70	1.20	3.00
Total carotenoid, μg/dL	11336	72.60	28.97	47.70	64.40	87.90	141.80
Dietary carotenoids							
α–Carotene, μg/day	10971	383.52	0.00	20.00	61.50	350.00	1840.00
β-Carotene, μg/day	10971	2017.06	95.50	374.00	922.00	2438.50	7233.50
β-Cryptoxanthin, μg/day	10971	141.60	0.50	9.50	40.00	176.00	571.00
Lycopene, μg/day	10971	6277.19	0.00	417.50	2634.50	8083.50	24271.00
Lutein/zeaxanthin, μg/day	10971	1455.95	107.00	387.00	757.00	1492.00	4922.00
Dietary antioxidant micronutrients							
Retinol, μg/day	10971	431.36	55.00	189.50	337.50	549.00	1035.50
Zinc, mg/day	10971	12.39	4.21	7.51	10.81	15.32	25.28
Selenium, mg/day	10971	110.86	41.20	72.80	100.45	136.90	212.95
Copper, mg/day	10971	1.34	0.52	0.88	1.19	1.59	2.51
Vitamin A, μg/day	10971	621.65	105.00	294.50	502.50	790.50	1494.50
Vitamin E, mg/day	10971	7.23	2.10	4.15	6.13	8.95	16.00
Vitamin C, mg/day	10971	89.92	8.10	29.40	62.95	120.90	257.60
Serum nutrient biomarkers							
Iron, μmol/L	11301	15.58	6.60	11.30	14.50	19.20	27.80
Folate, ng/mL	11308	13.82	5.30	8.50	11.80	16.30	28.30
Vitamin B12, pg/mL	11266	549.81	228.00	349.00	466.00	627.00	1020.00
Vitamin A, μg/dL	11336	60.52	36.60	49.10	59.00	70.00	88.80
Vitamin E, μg/dL	7717	1241.06	699.00	916.00	1140.00	1420.00	2150.00
Vitamin C, mg/dL	7014	0.95	0.17	0.61	0.94	1.23	1.75

N, number of urinary samples; 5th, 5th percentile; 25th, 25th percentile; 50th, 50th percentile; 75th, 75th percentile; 95th, 95th percentile.

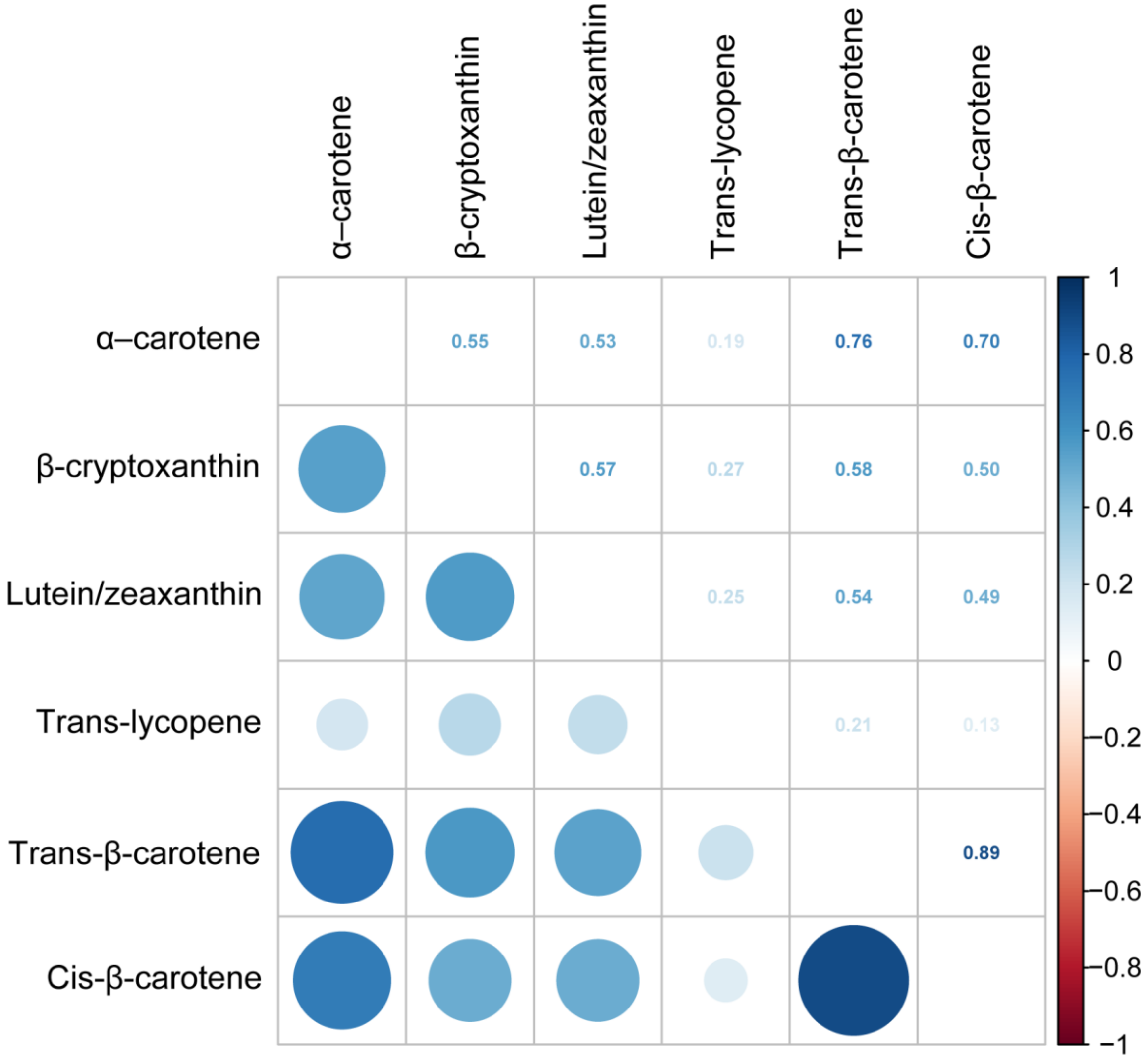

Supplemental Figure 2. Pairwise Pearson correlation coefficients between six serum carotenoids in adults.

Supplemental Table 2. OR (95% CIs) of hypertension (SBP≥140mmHg and/or DBP≥90mmHg) according to quartiles of serum carotenoids concentrations among the general adult population with further adjustment of dietary carotenoids and antioxidant micronutrients and serum nutrient biomarkers in NHANES 2001–2006.

Carotenoids	Quartiles of serum carotenoids (ug/dL)				P_{trend}
	Quartile 1	Quartile 2	Quartile 3	Quartile 4	
Model + dietary carotenoids *					
α-Carotene	1.00	0.82 (0.66-1.03)	0.71 (0.60-0.83)	0.60 (0.49-0.73)	<0.001
β-cryptoxanthin	1.00	0.80 (0.64-0.99)	0.68 (0.56-0.82)	0.66 (0.53-0.82)	<0.001
Lutein/zeaxanthin	1.00	0.91 (0.73-1.13)	0.91 (0.73-1.14)	0.85 (0.69-1.06)	0.157
Trans-lycopene	1.00	0.96 (0.78-1.18)	0.84 (0.71-1.01)	0.89 (0.70-1.13)	0.200
Trans-β-carotene	1.00	0.79 (0.68-0.93)	0.65 (0.52-0.80)	0.46 (0.37-0.57)	<0.001
Cis-β-carotene	1.00	0.80 (0.58-1.10)	0.62 (0.45-0.83)	0.46 (0.34-0.62)	<0.001
Model + dietary antioxidant micronutrients †					
α-Carotene	1.00	0.79 (0.63-0.99)	0.69 (0.57-0.83)	0.59 (0.48-0.73)	<0.001
β-cryptoxanthin	1.00	0.82 (0.65-1.04)	0.70 (0.58-0.85)	0.71 (0.57-0.89)	<0.001
Lutein/zeaxanthin	1.00	0.90 (0.72-1.13)	0.88 (0.70-1.11)	0.83 (0.66-1.04)	0.097
Trans-lycopene	1.00	0.93 (0.76-1.14)	0.83 (0.71-0.98)	0.91 (0.72-1.14)	0.237
Trans-β-carotene	1.00	0.77 (0.65-0.91)	0.60 (0.49-0.74)	0.41 (0.32-0.53)	<0.001
Cis-β-carotene	1.00	0.81 (0.59-1.11)	0.58 (0.43-0.80)	0.43 (0.30-0.59)	<0.001
Model + serum nutrient biomarkers ‡					
α-Carotene	1.00	0.81 (0.65-1.02)	0.70 (0.60-0.83)	0.60 (0.48-0.74)	<0.001
β-cryptoxanthin	1.00	0.81 (0.64-1.02)	0.68 (0.55-0.83)	0.68 (0.55-0.84)	<0.001
Lutein/zeaxanthin	1.00	0.91 (0.72-1.14)	0.90 (0.72-1.13)	0.84 (0.67-1.06)	0.137
Trans-lycopene	1.00	0.97 (0.79-1.19)	0.87 (0.74-1.02)	0.93 (0.74-1.16)	0.293
Trans-β-carotene	1.00	0.80 (0.68-0.94)	0.65 (0.52-0.81)	0.47 (0.38-0.59)	<0.001
Cis-β-carotene	1.00	0.79 (0.58-1.09)	0.61 (0.45-0.82)	0.46 (0.34-0.62)	<0.001

Model was adjusted for age, sex, education level, race, poverty, smoker, alcohol user, physical activity, energy intake levels, body mass index, hemoglobin, total cholesterol, high-density lipoprotein cholesterol, eGFR, and diabetes.

* Further adjusted for Model + dietary carotenoids (intakes of α-Carotene, β-Carotene, β-Cryptoxanthin, Lycopene, Lutein/zeaxanthin [all log2-transformed]).

† Further adjusted for Model + dietary antioxidant micronutrients (intakes of retinol, zinc, selenium, copper, vitamin A, vitamin E, vitamin C [all log2-transformed]).

‡ Further adjusted for Model + serum nutrient biomarkers (serum iron, folate, vitamin B12, vitamin A, vitamin E, and vitamin C levels [all log2-transformed]).

Supplemental Table 3. Multiple logistic regression associations of serum carotenoids with hypertension (SBP≥130mmHg and/or DBP≥80mmHg) in adults.

Carotenoids	Log2-carotenoids	Quartiles of serum carotenoids (ug/dL)				P_{trend}
		Quartile 1	Quartile 2	Quartile 3	Quartile 4	
α-carotene						
Model 1	0.81 (0.78-0.85)	Ref (1.00)	0.69 (0.59-0.81)	0.61 (0.52-0.71)	0.50 (0.41-0.60)	<0.001
Model 2	0.81 (0.77-0.85)	Ref (1.00)	0.70 (0.59-0.82)	0.61 (0.52-0.72)	0.49 (0.40-0.61)	<0.001
Model 3	0.87 (0.83-0.91)	Ref (1.00)	0.70 (0.58-0.85)	0.68 (0.58-0.79)	0.63 (0.52-0.79)	<0.001
β-cryptoxanthin						
Model 1	0.83 (0.78-0.88)	Ref (1.00)	0.84 (0.71-0.99)	0.65 (0.55-0.76)	0.58 (0.48-0.70)	<0.001
Model 2	0.81 (0.76-0.86)	Ref (1.00)	0.80 (0.67-0.95)	0.62 (0.52-0.73)	0.54 (0.44-0.67)	<0.001
Model 3	0.87 (0.82-0.93)	Ref (1.00)	0.85 (0.72-1.00)	0.69 (0.58-0.82)	0.69 (0.56-0.85)	<0.001
Lutein/zeaxanthin						
Model 1	0.78 (0.71-0.87)	Ref (1.00)	0.90 (0.76-1.06)	0.78 (0.66-0.93)	0.67 (0.54-0.82)	<0.001
Model 2	0.75 (0.67-0.84)	Ref (1.00)	0.88 (0.74-1.04)	0.75 (0.62-0.90)	0.63 (0.50-0.80)	<0.001
Model 3	0.80 (0.71-0.89)	Ref (1.00)	0.95 (0.79-1.14)	0.82 (0.68-0.99)	0.73 (0.58-0.92)	0.004
Trans-lycopene						
Model 1	0.97 (0.90-1.05)	Ref (1.00)	0.98 (0.83-1.15)	0.91 (0.77-1.08)	0.95 (0.79-1.14)	0.470
Model 2	0.98 (0.91-1.06)	Ref (1.00)	1.01 (0.85-1.19)	0.93 (0.78-1.11)	0.96 (0.80-1.16)	0.544
Model 3	0.91 (0.83-0.99)	Ref (1.00)	0.98 (0.82-1.17)	0.88 (0.73-1.06)	0.84 (0.69-1.02)	0.042
Trans-β-carotene						
Model 1	0.75 (0.71-0.79)	Ref (1.00)	0.67 (0.59-0.77)	0.59 (0.50-0.68)	0.37 (0.30-0.45)	<0.001
Model 2	0.73 (0.69-0.77)	Ref (1.00)	0.66 (0.58-0.76)	0.57 (0.48-0.66)	0.35 (0.28-0.43)	<0.001
Model 3	0.79 (0.75-0.84)	Ref (1.00)	0.72 (0.63-0.83)	0.66 (0.56-0.77)	0.46 (0.37-0.58)	<0.001
Cis-β-carotene						
Model 1	0.72 (0.67-0.77)	Ref (1.00)	0.85 (0.68-1.07)	0.66 (0.53-0.82)	0.42 (0.32-0.55)	<0.001
Model 2	0.70 (0.65-0.75)	Ref (1.00)	0.84 (0.67-1.06)	0.64 (0.51-0.79)	0.39 (0.30-0.52)	<0.001
Model 3	0.79 (0.73-0.85)	Ref (1.00)	0.88 (0.69-1.12)	0.73 (0.58-0.91)	0.52 (0.40-0.70)	<0.001

Model 1 was adjusted as age, sex;
Model 2 was adjusted as model 1 plus race, education levels, smoker, and alcohol user;
Model 3 was adjusted as model 2 plus physical activity, energy intake levels, body mass index, hemoglobin, total cholesterol, high-density lipoprotein cholesterol, eGFR, and diabetes.
Ref: reference; CI, confidence interval.

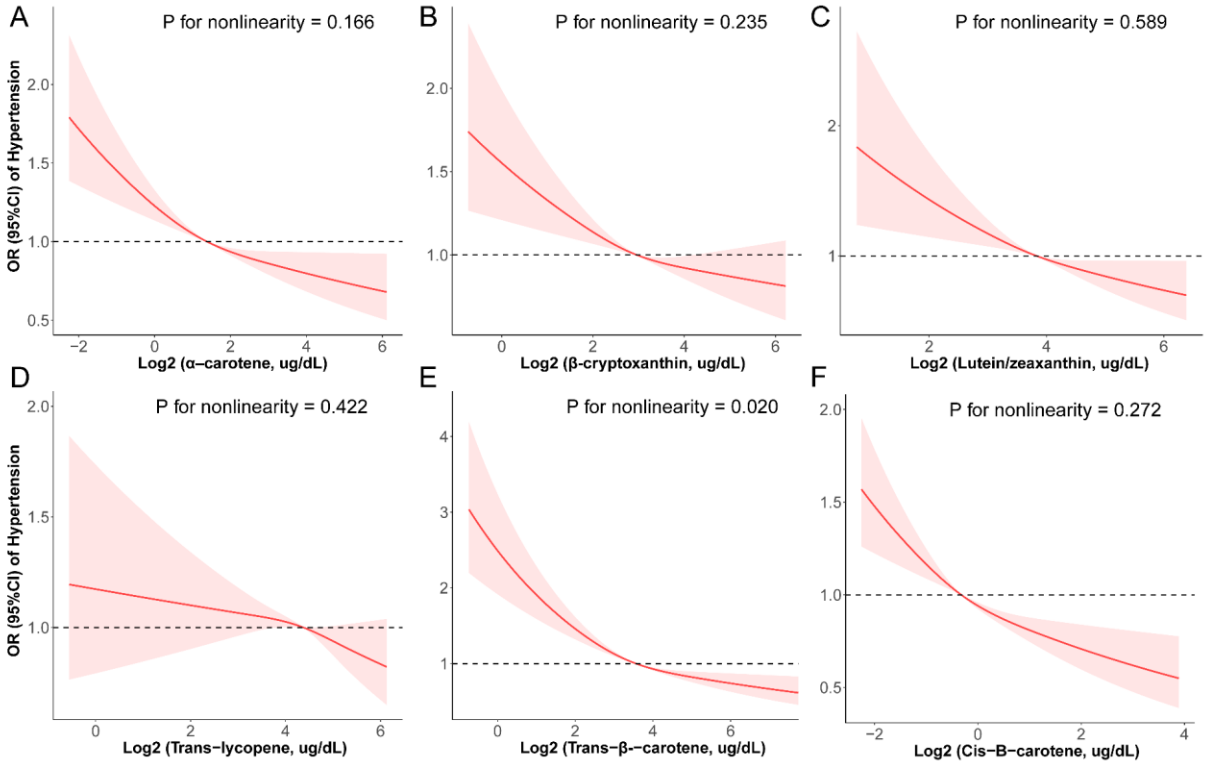

Supplemental Figure 3. Restricted cubic spline (RCS) analysis with multivariate-adjusted associations between six serum carotenoids and prevalence of hypertension (SBP≥130mmHg and/or DBP≥80mmHg) in adults. Models are adjusted for age, sex, education level, race, poverty, smoker, alcohol user, physical activity, energy intake levels, body mass index, hemoglobin, total cholesterol, high-density lipoprotein cholesterol, eGFR, and diabetes.
